# Improved Survival of HIV-1-Infected Patients with Progressive Multifocal Leukoencephalopathy Receiving Early 5-Drug Combination Antiretroviral Therapy

**DOI:** 10.1371/journal.pone.0020967

**Published:** 2011-06-30

**Authors:** Jacques Gasnault, Dominique Costagliola, Houria Hendel-Chavez, Anne Dulioust, Sophie Pakianather, Anne-Aurélie Mazet, Marie-Ghislaine de Goer de Herve, Rémi Lancar, Anne-Sophie Lascaux, Lydie Porte, Jean-François Delfraissy, Yassine Taoufik, for the ANRS 125 Trial Team

**Affiliations:** 1 Service de Médecine Interne et de Maladies Infectieuses, Hôpital Universitaire de Bicêtre - Assistance Publique Hôpitaux de Paris (APHP), Le Kremlin-Bicêtre, France; 2 INSERM U1012, Le Kremlin-Bicêtre, France; 3 Service de Maladies Infectieuses et Tropicales, Groupe hospitalier Pitié Salpêtrière - APHP, Paris, France; 4 INSERM U943, Paris, France; 5 UMR-S 943, UPMC Univ Paris 06, Paris, France; 6 Laboratoire d'Immunologie, Hôpital Universitaire de Bicêtre - APHP, Le Kremlin-Bicêtre, France; 7 Laboratoire de Virologie, Hôpital Universitaire de Bicêtre - APHP, Le Kremlin-Bicêtre, France; 8 Service d'Immunologie clinique, Hôpital Henri Mondor-APHP, Créteil, France; 9 Service des Maladies Infectieuses, Hôpital Purpan, Toulouse, France; 10 Faculté de Médecine de Bicêtre, UPS Univ Paris 11, Le Kremlin-Bicêtre, France; University of Toronto, Canada

## Abstract

**Background:**

Progressive multifocal leukoencephalopathy (PML), a rare devastating demyelinating disease caused by the polyomavirus JC (JCV), occurs in severely immunocompromised patients, most of whom have advanced-stage HIV infection. Despite combination antiretroviral therapy (cART), 50% of patients die within 6 months of PML onset. We conducted a multicenter, open-label pilot trial evaluating the survival benefit of a five-drug cART designed to accelerate HIV replication decay and JCV-specific immune recovery.

**Methods and Findings:**

All the patients received an optimized cART with three or more drugs for 12 months, plus the fusion inhibitor enfuvirtide during the first 6 months. The main endpoint was the one-year survival rate. A total of 28 patients were enrolled. At entry, median CD4+ T-cell count was 53 per microliter and 86% of patients had detectable plasma HIV RNA and CSF JCV DNA levels. Seven patients died, all before month 4. The one-year survival estimate was 0.75 (95% confidence interval, 0.61 to 0.93). At month 6, JCV DNA was undetectable in the CSF of 81% of survivors. At month 12, 81% of patients had undetectable plasma HIV RNA, and the median CD4+ T-cell increment was 105 per microliter. In univariate analysis, higher total and naive CD4+ T-cell counts and lower CSF JCV DNA level at baseline were associated with better survival. JCV-specific functional memory CD4+ T-cell responses, based on a proliferation assay, were detected in 4% of patients at baseline and 43% at M12 (P = 0.008).

**Conclusions:**

The early use of five-drug cART after PML diagnosis appears to improve survival. This is associated with recovery of anti-JCV T-cell responses and JCV clearance from CSF. A low CD4+ T-cell count (particularly naive subset) and high JCV DNA copies in CSF at PML diagnosis appear to be risk factors for death.

**Trial Registration:**

ClinicalTrials.gov NCT00120367

## Introduction

Human polyomavirus JC (JCV) is the causative agent of progressive multifocal leukoencephalopathy (PML), a rare demyelinating disease of the central nervous system (CNS) involving lytic infection of oligodendrocytes. PML occurs in severely immunocompromised individuals. The incidence of PML increased sharply during the HIV/AIDS pandemic, up until 1996 [1] and the advent of combination antiretroviral therapy (cART) era [2], [3]. It declined thereafter, but less slowly than the relative frequency of other AIDS-defining events [4]. Moreover, during the period 2001–2003, compared to the pre-cART era, the smallest decline in mortality observed in the French Hospital Database on HIV concerned patients with PML [4]. Recent results of the ART Cohort Collaboration confirm that PML, along with non-Hodgkin's lymphoma, remains associated with a higher risk of death than other AIDS-defining events [5].

T-cell responses likely play a key role in controlling intracerebral JCV replication. Indeed, specific cytotoxic CD8+ T-lymphocyte responses to JCV were found to be undetectable in patients who subsequently died, in contrast to survivors [6]. Help from CD4+ T-lymphocytes is required for optimal memory CD8+ T-cell responses during chronic viral infections [7]. We have previously reported the critical role of JCV-specific CD4+ T-cell responses in the control of JCV infection, and the lack of such responses in the early stages of active PML [8].

To date, no specific anti-JCV treatment has shown significant clinical efficacy in patients with PML [9]. Triple-drug ART has significantly improved the prognosis of HIV-associated PML [10]–[12], likely indirectly by restoring CD4+ T-cell responses to JCV [8]. However, about 50% of cART-treated patients still die within 6 months after PML clinical onset [9], [12], [13], while the remainders survive for more than a year [9], [14], [15]. In addition, most survivors have severe functional sequelae [9], [12], [15]. We wondered whether the early use of a more patent ART based on five-drug combination after PML onset could improve survival and neurological outcome, by accelerating the decay of HIV replication and, thus, JCV-specific T-cell restoration. The fusion inhibitor enfuvirtide, a 36-amino-acid peptide mimicking the HR2 region of HIV-1 gp41, prevents the completion of the HIV fusion sequence, thereby blocking cell entry. Enfuvirtide has been shown to bolster the potency of antiretroviral combinations in both naive and treatment-experienced HIV-infected patients [16]–.

We conducted a prospective multicenter study evaluating the effect of five-drug antiretroviral treatment including enfuvirtide on PML outcome.

## Methods and Patients

The protocol for this trial and supporting CONSORT checklist are available as supporting information (see Protocol S1 and Checklist S1).

### Study Design

The ANRS 125 trial, sponsored by the French National Agency for Research on AIDS and Viral Hepatitis (ANRS), was a multicenter open-label non comparative pilot study designed to assess the effect of an early use of five-drug cART including enfuvirtide on survival one year after PML diagnosis in HIV-1-infected patients. The study was designed to show an expected one-year survival rate of 70%, significantly higher than 45% which was the average of the one-year survival rate observed in previous reports [11]–[13], [15], [19]. A sample size of 24 subjects was chosen with the one-step Fleming test, with a power of 80% and a type-1 error of 5%. To compensate for invalid inclusions (up to 25%), it was decided to enroll 30 patients. The Institutional Review Board of Bicetre Hospital approved the study protocol and all clinical investigations conformed to the International Conference on Harmonization, Good Clinical Practice, and the Declaration of Helsinki. The Clinical.Trials.gov identifier is NCT00120367.

### Patients

All subjects had documented HIV type 1 (HIV-1) infection. All subjects were required to have a diagnosis of active PML, with less than 90 days of clinical progression [20], documented less than 30 days before study entry. Inclusion criteria were as follows: i) neurological findings compatible with focal lesions of the CNS; ii) one or more focal white-matter abnormalities, with no mass effect or enhancement, on cerebral magnetic resonance imaging (MRI), 3) no other likely cause; iv) detection of JCV DNA in CSF by polymerase chain reaction (PCR) or histopathological evidence of PML on brain biopsy. Written informed consent was obtained from each study participant or from next of kin if decision-making capacity was impaired. The exclusion criteria included age less than 18 years; pregnancy or breast-feeding; HIV-2 coinfection; concomitant opportunistic CNS infection; prior or concomitant immunotherapy (interleukin 2, alpha-interferon.); prior or concomitant treatment with cidofovir; prior treatment with enfuvirtide; and contraindications to enfuvirtide therapy. An independent committee validated all the diagnoses of PML.

### Study Treatments

All the participants received an effective cART for 12 months, plus enfuvirtide for the first 6 months. The choice of regimen was based on the individual ART history. Patients, who had never received ART when PML was diagnosed, received efavirenz, ritonavir-boosted lopinavir, and the fixed-dose emtricitabine-tenofovir combination. ART-experienced patients received drugs belonging to at least two different antiretroviral families, chosen on the basis of their treatment history and viral resistance genotyping results. Beyond 6 months, only patients with limited therapeutic options were maintained on enfurvirtide.

### Follow-up

Each participant was followed for one year. As the mean interval between the enrolment visit and the outset of enfuvirtide therapy was 1.4 days (range 0 to 11 days), the date of the first dose of enfuvirtide was chosen as the study baseline. The patients had physical examinations, including a standardized neurological examination and Karnosky performance-status measurement, at baseline, week 6 (W6), month 3 (M3) and month 6 (M6). Karnosky performance-status and the modified Rankin scale (MRS) [21] were assessed at month 12 (M12). Cerebral MRI was performed at baseline and between W6 and M6, in case of clinical progression. Blood samples for HIV RNA assay, lymphocyte phenotyping and assessment of JCV-specific T-cell responses were collected at baseline, W6, M3, M6 and M12. Lumbar puncture for HIV RNA and JCV DNA assay in CSF was performed at baseline, M3 and M6.

#### Neurological scores

We used a standardized neurological examination from the NIH Stroke Scale [22], adapted for PML (Table S1). Briefly, this scale includes an assessment of consciousness, motor and sensory functions, coordination, cranial nerve functions, phonation, swallowing, visual fields, and cognition (language, memory and executive functions). A summary score ranging from 0 (normal) to 60 (very abnormal) was determined for each examination.

#### Laboratory Methods

JCV DNA was quantified in CSF by using a quantitative real-time PCR method, [23]. Briefly, DNA was extracted from 200 microliter of CSF with the QIAamp DNA blood kit (QIAGEN GmbH, Hilden, Germany), according to the manufacturer's protocol. A 113-bp fragment of the open reading frame coding for the large T antigen was amplified with the JCV primers PEP3 (5′ gga aag tct tta ggg tct tct acc ttt 3′) and PEP6 (5′ gaa gac cgt ttt tgc cat gaa ga 3′). PCR product was detected with the HuPo1 probe (5′ atc acg ggc aaa cat 3′). To control for the presence of PCR inhibitors, an internal DNA control (DICO Ampli r-gene; Argene, Varilhes, France) was simultaneously amplified in each PCR run. The detection limit was 100 copies per milliliter.

Flow cytometry used a FACsCalibur device (Becton Dickinson, Rungis, France), with monoclonal antibodies and reagents provided by the same manufacturer. T-cells subsets are defined by the following antibody expression patterns: Naive subset, CD45RA+CD62L+; Central memory subset, CD45RA−CD62L+; Effector memory subset, CD45RA−CD62L−; Effector subset, CD45RA+CD62L−.

JCV-specific CD4+ T-cell responses were assessed in proliferation assays with purified JCV (MAD-4 strain) [8]. In each experiment a proliferation index (PI) was determined as the ratio of median counts per minute (cpm) of activated wells (in quadriplicate) to unstimulated wells (containing no antigen). CD4 T-cell proliferation to JCV was considered significant if the following conditions were met: (i) median PI>3, (ii) median cpm of activated wells >2500, and (iii) no significant proliferation of CD4 T-cell-depleted PBMC in response to JCV. Positive control wells contained phytohemagglutinin (PHA) or Staphylococcal Enterotoxin B (SEB).

To investigate anti-JCV effector T-cell responses, we used an *ex vivo* interferon-gamma Elispot assay with PBMC and 14 pools of overlapping 15-amino-acid peptides covering the entire JCV VP-1 protein. In some patients, the effect of JCV peptide pools on T-cell responses was also examine by flow cytometry, the interferon-gamma responses mainly involved CD8 T-cells. The pools were used at 2 µg per milliliter. Positive controls used PMA (50 ng per milliliter) and ionomycin (500 ng per milliliter). The response to each pool was considered positive if the mean number of spot-forming cells (SFC) per 10^6^ PBMC in activated wells (in triplicate) was more than 2-fold that of untreated wells (background) and also higher than the background plus 100 SFC. A patient was considered to be a responder, i.e. to have a detectable anti-JCV effector T-cell response, if he or she had a positive response to at least one peptide pool.

#### Statistical considerations

The primary endpoint was the one-year survival rate. Secondary endpoints were the percentage of patients who survived at M12 with a MRS score below 3 (slight disability, independent for activities of daily-living); the percentage change in neurological scores between baseline and M6; the change in Karnofsky performance-status between baseline and M12; the change in CSF JCV level and the proportion of patients with CSF JCV clearance at M6; and the changes in CD4+ and CD8+ T-cell subsets and in JCV-specific T-cell responses between baseline and W6, M3, M6 and M12. Non-parametric methods (Wilcoxon's signed-rank test, Mann-Whitney test, Fisher's two-sided exact test, and McNemar's test) were used to compare continuous and categorical variables. Survival rates were estimated with the Kaplan-Meier method from the first dose of enfuvirtide. Survival curves were compared with the log-rank test.

## Results

Between April 2005 and December 2006, 29 patients were enrolled in six centers, including 21 patients in the main center (Bicêtre Hospital, Paris area). One patient subsequently withdrew consent, and the study population therefore consisted of 28 patients.

### Characteristics at entry

Baseline clinical and immunovirological data are shown in Table 1. The median interval between neurological symptom onset and study entry was 46 days (range, 11 to 88). The 12 patients who had never received antiretroviral drugs did not differ significantly from the 16 previously treated patients, with the exception of a lower plasma HIV RNA value in the latter (Mann-Whitney test, P = 0.017). At inclusion, all four patients with plasma HIV RNA below 40 copies/mL had positive JCV PCR in CSF. These patients had started an ARV treatment for less than 2 months before inclusion.

**Table 1:** Characteristics of patients at enrolment, according to outcome.

Variables	Total (n = 28)	Alive (n = 21)	Deceased (n = 7)	P-values
**ART naive (#)**	12	8	4	0.42
**Prior AIDS (#)**	9	8	1	0.37
**Gender** (men∶ women)	22∶ 6	15∶ 6	7∶ 0	0.29
**Age** *(years)*	42 (35–48)	42 (36–49)	42 (35–46)	0.71
**Time before study entry** *(days)*	46 (32–69)	46 (35–71)	31 (21–59)	0.22
**Neurological Score**	7.0 (5.0–10.8)	7.0 (5.0–10.5)	7.0 (3.0–15.0)	0.89
**Karnofsky Performance-Status** *(%)*	60 (40–70)	50 (40–70)	60 ‘40–70)	0.75
**Total CD4+ T cells** *(per µL)*	**53 (19–157)**	**73 (24–242)**	**26 (13–32)**	**0.03**
**Total CD8+ T cells** *(per µL)*	607 (422–1127)	741 (441–1153)	459 (409–631)	0.16
**CD4∶CD8 ratio**	0.09 (0.02–0.20)	0.15 (0.03–0.28)	0.05 (0.02–0.09)	0.05
**Naive CD4+ T cells** *(per µL)*	**2 (1–37)**	**5 (1–51)**	**1 (0–1)**	**0.006**
**Central memory CD4+ T cells** *(per µL)*	19 (8–69)	29 (10–76)	14 (2–19)	0.06
**Effector memory CD4+ T cells** *(per µL)*	14 (4–40)	18 (4–42)	11 (8–18)	0.32
**Naive CD8+ T cells** *(per µL)*	97 (53–169)	112 (66–212)	53 (26–134)	0.10
**Central memory CD8+ T cells** *(per µL)*	97 (49–160)	98 (47–162)	83 (49–135)	0.89
**Effector memory CD8+ T cells** *(per µL)*	174 (96–492)	167 (97–516)	191 (78–423)	0.96
**Effector CD8+ T cells** *(per µL)*	174 (70–326)	265 (73–366)	123 (45–189)	0.13
**Plasma HIV RNA** *(log10 copies/mL)*	4.0 (2.5–4.9)	3.6 (2.4–4.9)	4.2 (3.1–4.8)	0.60
**CSF HIV RNA** *(log10 copies/mL)*	2.3 (2.2–4.0)	2.5 (2.0–4.4)	2.3 (2.3–2.3)	0.56
**CSF JCV DNA** *(log10 copies/mL)*	**2.6 (2.0–3.2)**	**2.5 (1.3–3.1)**	**3.2 (2.5–4.8)**	**0.04**
**CSF JCV DNA** *(% >100 copies/mL)*	85.7% [24/28]	80.9% [17/21]	100% [7/7]	0.55*

**Note:** Median values are shown, with interquartile range in parentheses. P-values are not adjusted for multiple comparisons. ART: antiretroviral therapy. CSF: cerebrospinal fluid. T cells subsets are defined by the following antibody expression patterns: Naive subset, CD45RA+CD62L+; Central memory subset, CD45RA−CD62L+; Effector memory subset, CD45RA−CD62L−; Effector subset, CD45RA+CD62L−. (µL = microliter, mL = millimeter).

All the patients received enfuvirtide until death or for at least 6 months. Five patients continued to receive enfuvirtide after 6 months, for an average total period of 11 months (range, 7 to 18). In addition to enfuvirtide, 15 patients, including all 12 previously untreated patients, received efavirenz, ritonavir-boosted lopinavir and tenofovir/emtricitabine. The other 13 patients received other combinations consisting of 2 or 3 reverse transcriptase inhibitors (tenofovir 9, emtricitabine 7, abacavir 4, lamivudine 4, didanosine 3, efavirenz 2, zidovudine 1) plus 1 or 2 ritonavir-boosted protease inhibitors (fosamprenavir 8, lopinavir 7, saquinavir 2, indinavir 2). Percutaneous endoscopic gastrostomy tube for enteral feeding and drugs administration was inserted in 7 patients with swallowing disorders.

### Survival

**Figure 1:**
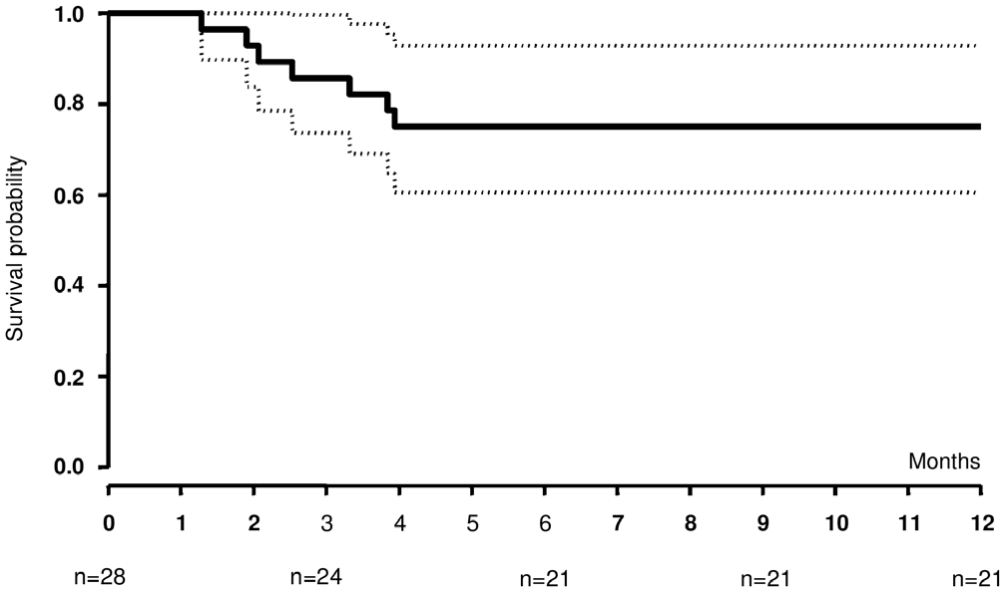
Survival of PML patients on five-drug antiretroviral treatment. Kaplan-Meier estimates and 95% confidence interval of the survival rate during five-drug antiretroviral therapy after PML diagnosis. Baseline is the date of the first dose of enfuvirtide. Follow-up is censored at 12 months. The one-year cumulative probability of survival was 0.75 (95% CI, 0.61–0.93). Seven patients died, all before 4 months.

Seven patients died during the study period, all from PML, and all before M4 including one with plasma HIV RNA below 40 copies/mL. The estimated one-year survival rate was 0.75 (95% confidence interval, 0.61 to 0.93) (Figure 1). No significant difference in terms of survival rate was observed between patients enrolled in the main center and the other patients (0.80 vs. 0.63; log-rank test, P = 0.31). The survival rate did not differ significantly between naïve and previously treated patients (0.67 vs. 0.81; log-rank test, P = 0.44).

### Changes in neurological and performance scores

**Figure 2.**
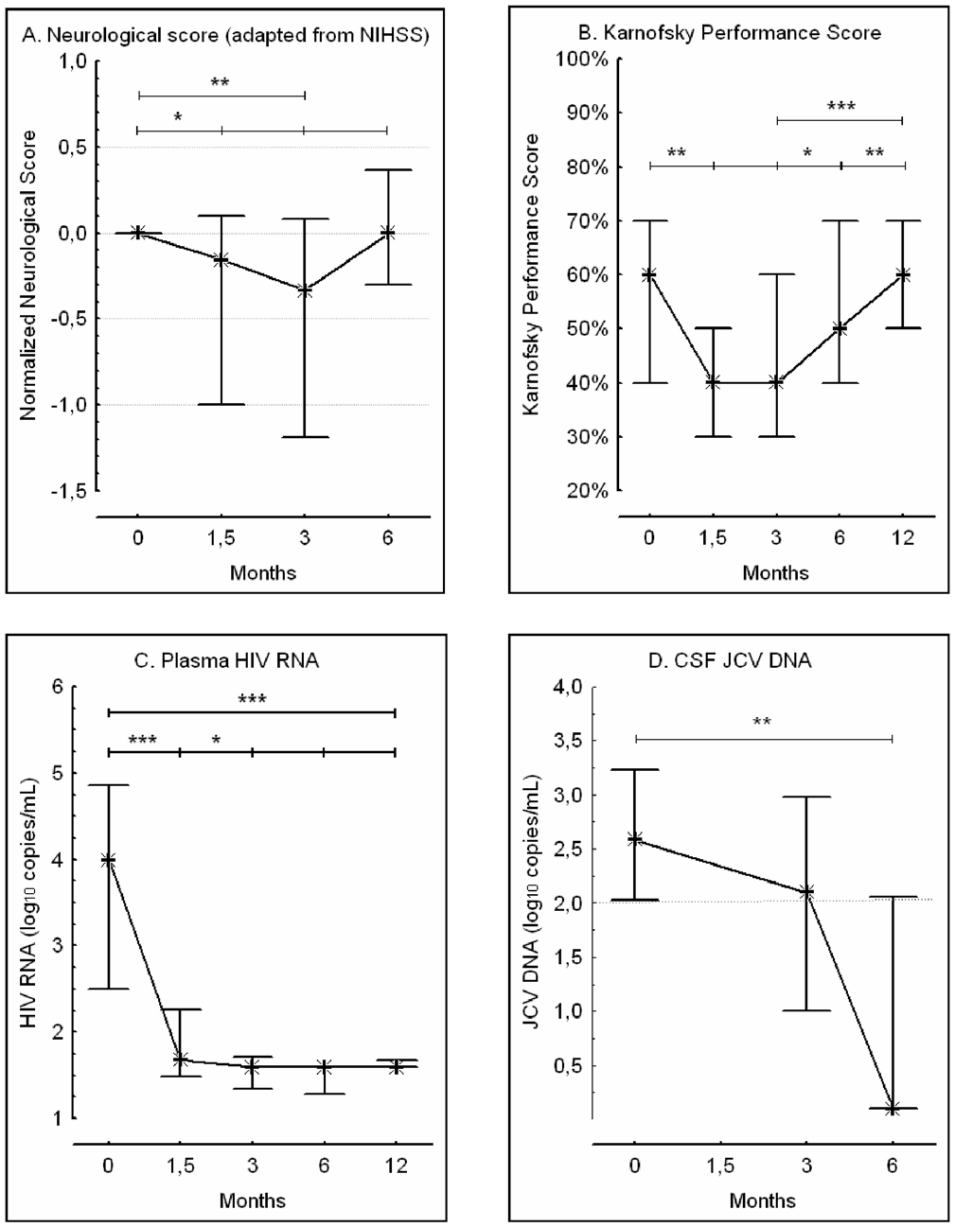
Time course of clinical and virological parameters. Biphasic course of a normalized neurological score from baseline to M6 (panel A) and of the Karnofsky performance score from baseline to M12 (panel B). Early and sustained plasma HIV RNA response from baseline to M12 on early five-drug antiretroviral therapy (panel C). Significant reduction in JCV DNA in CSF at M6 (panel D). Whiskers represent medians and interquartiles. Wilcoxon's signed-rank test : * P<0.05, ** P<0.01, *** P<0.001.

As shown in Figure 2 (panels A and B), the clinical course was biphasic. Compared to baseline (Table 2), the standardized neurological score worsened significantly at W6 and at M3 (Wilcoxon's signed-rank test, respectively P = 0.02 and P = 0.008). By contrast, it tended to improve at M6 as compared to M3 (P = 0.10), and was no longer significantly different from baseline (P = 0.71). Similarly, compared to baseline, Karnofsky performance-status deteriorated significantly at W6 (P = 0.002) and M3 (P = 0.02), before improving at M6 (P = 0.03) and M12 (P = 0.001) relative to M3. At M6 and M12, Karnofsky performance-status was no longer significantly different from baseline (respectively, P = 0.57, and P = 0.34). At M12, the 21 survivors had a median MRS of 3 (range, 1 to 5). Eight patients (38%) had no more than slight disability and were independent for activities of daily living (MRS<3), 9 patients (43%) were moderately disabled (MRS = 3), and 4 patients (19%) had moderately severe to severe disability (MRS>3).

**Table 2:** Changes from baseline characteristics.

Variables	Week 6	Month 3	Month 6	Month 12
**Standardized Neurological Score**	−0.2 (−1.0–0.1)	−0.3 (−1.2–0.1)	0.0 (−03–0.4)	
**Karnofsky Performance-Status**	−10 (−20–0)	−10 (−30–5)	0 (−20–10)	0 (−10–10)
**Total CD4+ T cells**	24 (0–58)	35 (7–86)	66 (45–117)	105 (18–127)
**Total CD8+ T cells**	80 (−42–336)	45 (−111–365)	69 (−130–328)	111 (−70–327)
**Naive CD4+ T cells)**	2 (−0.2–5)	2 (0–9)	12 (3–34)	29 (24–54)
**Central memory CD4+ T cells**	7 (1–34)	11 (0.2–40)	22 (5–41)	49 (11–61)
**Effector memory CD4+ T cells**	7 (−0.1–21)	8 (2–24)	12 (5–24)	10 (−0.1–21)
**Naive CD8+ T cells**	21 (−9–37)	27 (−26–60)	26 (10–85)	69 (17–135)
**Central memory CD8+ T cells**	−5 (−19–31)	−16 (−38–11)	−8 (−46–28)	8 (−31–72)
**Effector memory CD8+ T cells**	34 (−25–150)	−0.2 (−85–126)	3 (−72–36)	14 (−58–169)
**Effector CD8+ T cells**	7 (−49–59)	12 (−74–79)	18 (−29–116)	11 (−35–125)

**Note:** Median change relative to baseline is shown, with interquartile range in parentheses. T cells subsets are defined by the following antibody expression patterns: Naive subset, CD45RA+CD62L+; Central memory subset, CD45RA−CD62L+; Effector memory subset, CD45RA−CD62L−; Effector subset, CD45RA+CD62L−. Values of T cells (total and subsets) are cells per microliter.

### Viral kinetics

Treatment was rapidly and persistently effective on plasma HIV RNA level (Figure 2 panel C): values were below 50 copies per milliliter in 14% of patients (4/28) at baseline, 50% (13/26) at W6, 75% (18/24) at M3, 86% (18/21) at M6, 90% (19/21) at M9 and 81% (17/21) at M12. JCV DNA level in CSF also fell from baseline to M6 (Figure 2 panel D): values were below 100 copies per milliliter in 14% of patients (4/28) at baseline, 40% of patients (10/25) at M3 (Fisher's exact test, P = 0.007) and 81% of patients (17/21) at M6 (P<0.001).

### Immunological changes

**Figure 3:**
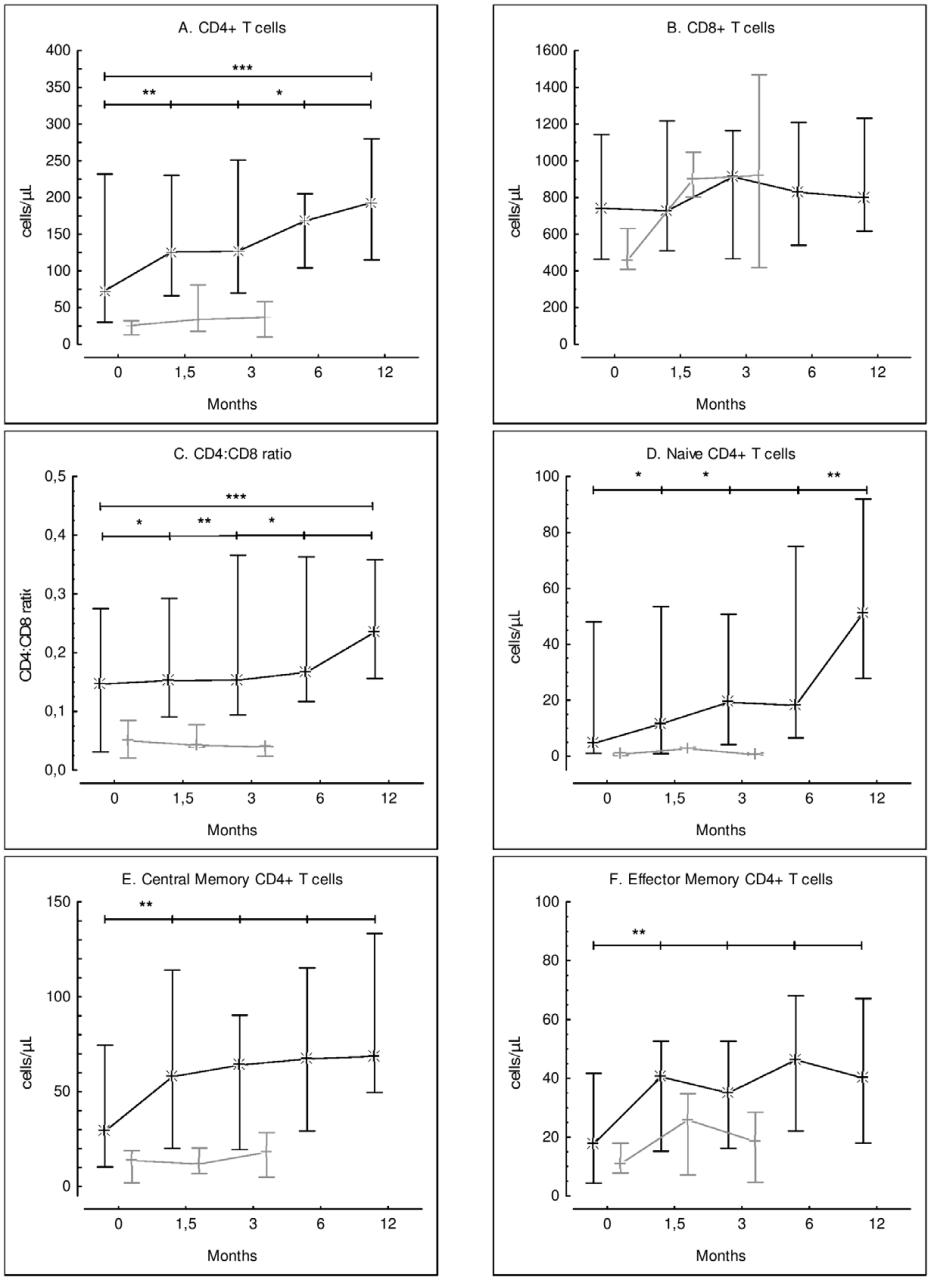
Time course of CD4+ and CD8+ T cell populations from baseline to month 12. Compared to baseline, significant increases were observed at W6 and at each subsequent time point in: the CD4+ T cell count (panel A); the CD4∶CD8 ratio (panel C); naive CD4+ T cells (panel D); central memory CD4+ T cells (panel E); and effector memory CD4+ T cells (panel F). No significant variation in the CD8+ T cell count (panel B) was noted during follow-up. (naive = CD45RA+CD62L+, central memory = CD45RA−CD62L+), effector memory = CD45RA−CD62L−). Whiskers represent medians and interquartiles. Black lines represent survivors and grey lines patients who died. Wilcoxon's signed-rank test : * P<0.05, ** P<0.01, *** P<0.001.

See Table 2. The CD4+ T-cell count increased significantly from baseline to W6 (P = 0.003), and at each subsequent time point (Figure 3 panel A). At M12, the median CD4+ T-cell increment was 105 per microliter (IQR, 18 to 127). In contrast, the CD8+ T-cell count did not change significantly during follow-up (Figure 3 panel B). As shown in Figure 3 (panel C), the CD4∶CD8 ratio increased significantly at each time point vs. baseline, with the exception of M12 compared to M6 (P = 0.13).

The naive CD4+ T-cell count (Figure 3 panel D) increased significantly from baseline to W6 (P = 0.02) and from W6 to M3 (P = 0.02), while it remained stable between M3 and M6 (P = 0.50) and increased markedly from M6 to M12 (P = 0.005). Central memory and effector memory CD4+ T-cell numbers increased significantly from baseline to W6 (respectively, P = 0.01 and P = 0.005), with no significant variations thereafter (Figure 3 panels E and F).

Naive CD8+ T-cell numbers increased only from W6 to M3 (P = 0.05) (not shown). No significant change was observed in the numbers of central memory, effector memory or effector CD8+ T-cells.

**Figure 4:**
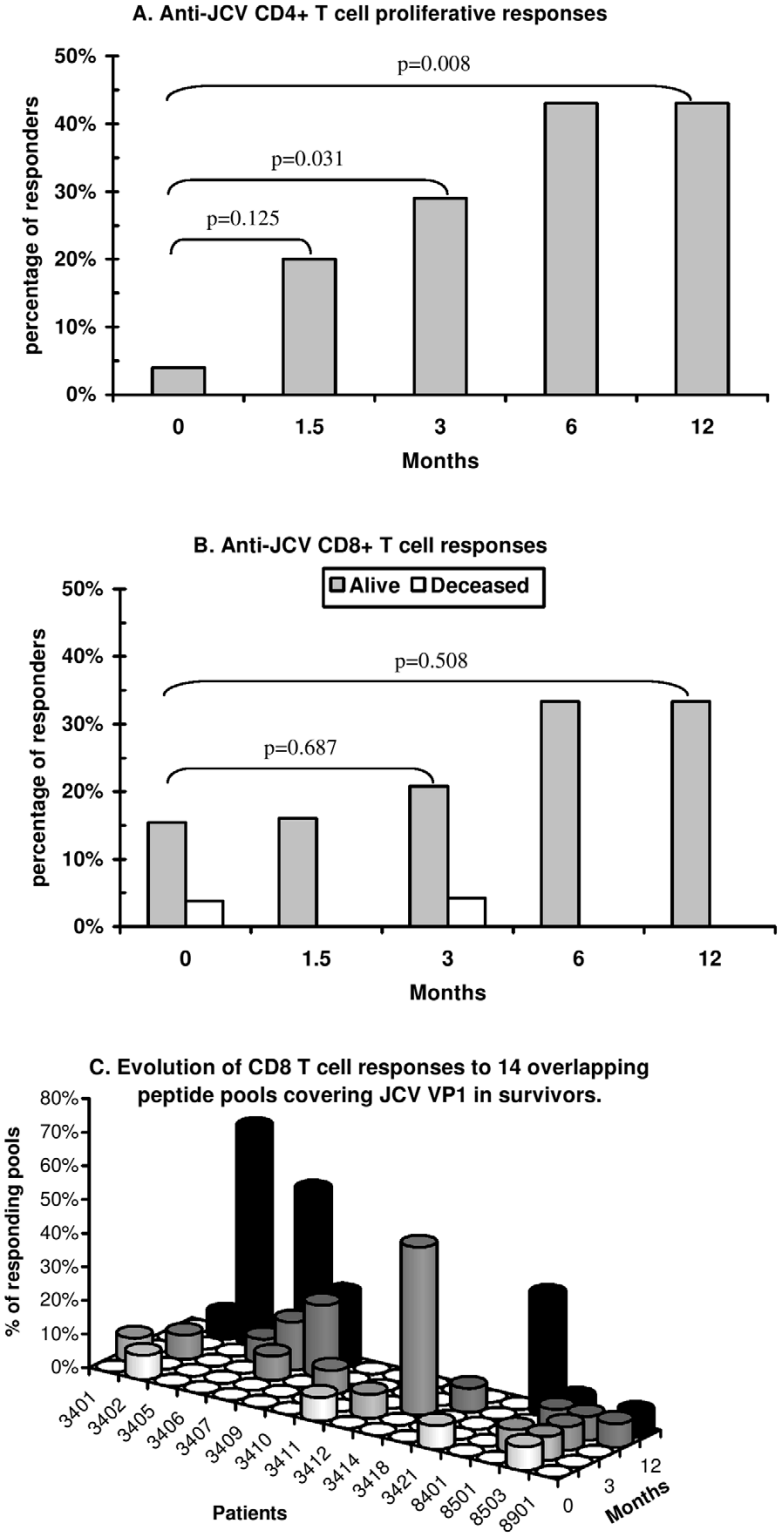
JCV-specific T-cell responses during follow-up. Panel A: Analysis of anti-JCV CD4+ T cell responses. Panel B: Analysis of *ex-vivo* anti-JCV CD8+ T-cell responses. A patient was considered to be a responder if he or she had a positive response to at least one JCV VP1 overlapping peptide pool among the 14 pools tested. Panel C shows the percentage of responding pools among the 14 pools tested in each survivor during follow-up. Five of the 21 survivors who had no responses at any time are not represented.

Significant restoration of anti-JCV CD4+ T-cell proliferation to purified JCV was observed, starting at M3 (Figure 4 panel A). The percentage of “responder” patients increased from 4% at baseline to 29% at M3 (McNemar's test, P = 0.03) and to 43% at M6 and M12 (P = 0.008).

Using an *ex vivo* interferon gamma Elispot assay and overlapping peptide pools covering the entire VP1 protein to test effector T-cell responses including mainly CD8+ T-cell responses, we found no significant changes in the percentage of patients responding to at least one peptide pool between baseline (19%) and M12 (33%, P = 0.51) or any other time during the follow-up (Figure 4 panel B). We then analyzed the range of effector T-cell responses during follow-up. At baseline and W6, four patients responded to a single peptide pool (mean of responding pools = 0.19 in the 21 survivors). At M3, the mean number of responding pools was 2.20 and 0.52 in responders and in all survivors, respectively. The respective mean numbers were 1.43 and 0.48 at M6 vs. 3.86 and 1.29 at M12. Among the 21 survivors, paired analysis showed a trend towards a larger number of responding peptide pools at M12 as compared to baseline (Wilcoxon's signed-rank test, P = 0.10) (Figure 4 panel C). Interestingly, in a given patient, anti-JCV effector T-cell responses were variable during follow-up (Figure 4 panel C).

### Factors associated with survival

See Table 1. Patients who died had lower baseline peripheral CD4+ T-cell counts than survivors and tended to have lower CD4∶CD8 ratios. The naive CD4+ T-cell count at baseline was significantly lower in patients who died than in survivors. Central memory CD4+ T-cell numbers tended to be lower at baseline in patients who died than in survivors. Likewise, patients who died tended to have lower baseline naive CD8+ T-cell counts than survivors and lower effector CD8+ T-cell counts. Moreover, baseline CSF JCV DNA levels measured in 27 patients were significantly higher in patients who died than in survivors.

The time between clinical onset and study entry, the baseline neurological examination score, Karnofsky performance-status and HIV RNA levels in plasma and CSF were not significantly different between patients who died and those who survived. Furthermore, activity level of antiretroviral drugs into the CNS, evaluated by the 2010-revised CNS Penetration Effectiveness Score [24], did not differ significantly between survivors and non survivors (Mann-Whitney test, P = 0.914).

In summary, higher counts of total and naive CD4+ T-cells at baseline, along with lower JCV DNA levels, were associated with better survival. Among baseline immunological markers, a higher CD4∶CD8 ratio, a higher central memory CD4+ T-cell count and a higher naive CD8+ T-cell count tended to be associated with better survival.

JCV-specific CD4+ T-cell proliferative responses were detected at least once during follow-up in 52% (11/21) of survivors and in none of the 7 patients who died (Fisher's exact test, p = 0.023), while specific effector T-cell responses were found in 76% (16/21) of survivors and in 29% (2/7) of patients who died (P = 0.06).

Moreover no correlation was observed between JCV viral load in CSF and JCV-specific T-cell responses both at baseline and during follow-up (M3 and M6). Finally no relationship has been found between neurological outcome (evaluated by MRS) and JCV-specific T-cell responses at M12 as well as JCV viral load during follow-up.

### Safety

A possible immune restoration inflammatory syndrome (IRIS) occurred in a treatment-naive patient with a baseline CD4+ T-cell count of 167 per microliter, whose neurological status markedly deteriorated (right hemiplegia, aphasia, double hemianopia and dysphagia) during the first 6 weeks of ART. At W6, the CD4+ T-cell count was 214 per microliter and cerebral MRI showed extension of demyelination to the white matter of both hemispheres, including the corpus callosum. A mass effect on the left ventricle and perilesional contrast enhancement were also observed. The patient improved rapidly on steroid therapy. Of note, this patient had no detectable specific CD4+ T-cell responses during the study period, and a positive anti-JCV effector T-cell response was observed only at M12.

Six serious adverse events occurred in 6 different patients, consisting of one case each of bicytopenia, bilateral pulmonary embolism, partial epilepsy, acute respiratory distress syndrome, biochemical deterioration of hepatitis C, and hysterectomy for severe cervical dysplasia. With the exception of bicytopenia, these serious adverse events were assessed as not directly related to the antiretroviral therapy. All serious events resolved, except in the patient with severe hepatitis C, whose liver status continued to deteriorate despite interruption of ART and who died at week 6. All other patients were still alive at the end of the study. Enfuvirtide was never withdrawn. Urolithiasis occurred in a patient on indinavir, which was replaced by atazanavir. No significant variations in creatinine clearance, calcemia or phosphatemia occurred in the 24 patients receiving tenofovir.

## Discussion

In this study the one-year cumulative probability of survival after PML diagnosis was 75% (95% confidence interval, 0.61 to 0.93), a rate higher than previously reported in HIV-infected patients placed on conventional triple-drug ART after PML diagnosis (39% to 56%) [10]–[15], [19], [25]. This suggests that a five-drug antiretroviral regimen given early after PML diagnosis may improve survival. With a five-drug combination, none of our patients died after four months. However, this treatment remains insufficient for patients with very rapidly progressive disease. As a result, this study also showed a 4-month survival rate of 75%. In previous reports concerning standard cART [13], [20], most PML-related deaths occurred during the first months with a median time of 1.6 to 4.3 months following PML diagnosis. In the Danish HIV Cohort Study, 35 of the 47 patients, diagnosed with PML between 1995 and 2006, died during the study period, including 18 (38.3%) whose death occurred within 4 months after the onset of PML [3]. In a more recent study of patients diagnosed with PML between 2002 and 2006, the probability of survival was 61% (95% CI, 48 to 72) at 3 months, 48% (95% CI, 35 to 59) at 6 months and 39% (95% CI, 25 to 51) at 12 months [25].

There are few longitudinal studies of the clinical course of PML during cART. In a previous paper [12], we showed no significant change in neurological assessment based on the EDSS score between the last time point (median 539 days) and cART initiation. On the MRS, 38% of the survivors in our study had, at most, slight disability, as compared to 31% of 42 historical control patients [9]. By contrast, the odds of having moderately severe to severe disability 12 months after PML diagnosis were lower (19%) than in a multicohort study of 370 patients with PML placed on conventional cART (39%) [15].

The low incidence of HIV-related PML led us to choose an open-label non comparative study design. To demonstrate in a randomized controlled study that a one-year survival rate of 70%, was significantly higher than 45% which was the average of the survival rate observed in previous published studies [11]–[13], [15], [19], it would have been necessary to enroll 128 patients. These studies included patients treated with standard triple-drug antiretroviral combinations mainly between January 1996 and February 2004. Our patients were similar at PML diagnosis to the patients described in these five historical series and in three more recent reports [3], [14], [25], with respect to proportion of ARV-naïve patients, CD4 T-cell count, plasma viral load (for more information, see Table S2).

Use of triple-drug cART in patients with AIDS-related PML can cause IRIS [19], [25]–[27]. The use of five-drug cART could potentially augment the frequency and severity of PML-IRIS. In our study, one possible case of IRIS was observed, 6 weeks after initiation of the study treatment in a previously ART-naive patient. The diagnosis of IRIS was based on clinical deterioration and on MRI evidence of inflammatory modifications of PML lesions. The clinical status of PML patients frequently keeps on deteriorating during the first weeks of ART, possibly owing to the delay in anti-JCV immune recovery (see Fig. 2). In our patient, perilesional contrast enhancement and a mass effect on the left ventricle, observed on MRI, supported the diagnosis of IRIS. Steroids were rapidly effective, as in a retrospective study of a large cohort of patients with IRIS-associated PML [26]. In our study, the upper limit of the 95% confidence interval of IRIS occurrence (1/28) was 18%. In comparison, PML-associated IRIS have been reported in 18% to 23% of patients in the setting of standard triple-drug ART [19], [25], [26]. Thus, five-drug combination does not seem to increase the risk of PML-associated IRIS.

In this study, survival of PML patients was associated with a high rate of JCV clearance from CSF, as previously reported [9], [10], [12], [28]. Combined antiretroviral therapy may act on intracerebral JCV replication by reducing HIV replication and inflammation in the brain. A transactivating effect of HIV Tat protein on JCV promoters has also been reported [29]. Blood-brain barrier modifications due to HIV infection may favor the entry of JCV-harboring B cells, while local cytokine production may enhance JCV replication [1]. However, the main mechanism by which ART acts on JCV replication is likely to be the recovery of specific anti-JCV responses. Indeed, we observed significant recovery of specific CD4+ T-cell proliferative responses from M3 to M12, and this was associated with better survival, in line with the result of a cross-sectional study [8]. Among memory CD4+ T-cells, proliferation is mainly the hallmark of central memory cells, which are also associated with CD4 help to memory CD8+ T-cells [7], [30], [31]. Several lines of evidence show the critical role of CD4 help for the maintenance of effective secondary cytotoxic CD8+ T-cell responses [7], [32], [33]. Of interest, at baseline, the number of circulating naive CD4+ T-cells and, to a lesser extent, the number of central memory CD4+ T-cells, were higher in PML patients who survived. The presence of a larger residual pool of central memory and naive CD4+ T-cells in subsequent survivors may accelerate the return of an effective JCV-specific memory CD4+ T-cell response during five-drug cART. This could involve some functional recovery of JCV-specific memory cells, and improved generation of memory CD4+ T-cells from the residual naive pool following effective inhibition of HIV replication. The existence of only small residual pool of naive CD4+ T-cells might therefore be a limiting factor in the immune control of intracerebral JCV replication, given the slow expansion of these cells during effective cART (Fig. 3D).

Better outcome of PML has been linked to the detection of anti-JCV peripheral blood CD8+ CTL after peptide stimulation *in vitro* for 10 to 14 days [6], [34]. Here, we used an *ex vivo* procedure, without *in vitro* amplification, to assess circulating IFN-gamma-producing anti-JCV T-cell effectors including mainly CD8+ effectors. Survival was not associated with the detection of anti-JCV T-cell effectors at baseline, but most JCV-specific T-cell effectors might be located in brain tissue, where JCV replication occurs predominantly during active PML. This, in addition to the inconstancy of detection of anti-JCV T-cell effectors in a given patient during follow-up, would mean that the blood compartment does not accurately reflect anti-JCV T-cell responsiveness. This is consistent with recent studies based on the use of specific tetramers, that showed a very low frequency of anti-JCV specific CD8+ T-cells in peripheral blood, relative to infections by less anatomically restricted viruses such as HIV, EBV and CMV [35]. Moreover, while a drastic fall in CSF JCV DNA level occurred in most of our patients at M6, the percentage of patients whose peripheral cells responded in the Elispot assay did not increase during follow-up. However, at M12, a trend towards a broader repertoire of circulating anti-JCV T-cell responses was observed. This emergence of additional anti-JCV specificities in blood could result from several mechanisms, including redistribution of CD8+ T-cells previously trapped in brain tissue, generation of new anti-JCV memory CD8+ T-cells, and improved function of pre-existing memory cells. The last two mechanisms are dependent on help from anti-JCV CD4+ T-cells, which expanded during ART.

By accelerating HIV decay, five-drug antiretroviral combination may lead to more rapid anti-JCV immune reconstitution. However, 25% of patients still die within 4 months of treatment initiation. Moreover, only 38% of survivors recovered adequate autonomy for activities of daily living. The size of the residual naive and central memory CD4+ T-cell pools may influence the time required for effective CD8-helping CD4+ T-cell responses to recover during cART. These pools could therefore represent targets for immunotherapy based on cytokines such as IL-7 [36].

In conclusion, our results support the use of five-drug cART early during the course of PML, for both antiretroviral-naive and -experienced patients.

## Supporting Information

Table S1 **The PML Score is a clinical assessment tool adapted from the NIH Stroke Scale (NIHSS, Brott et al. Stroke 1989).** To evaluate and document neurological status in PML patients, four items (vestibular function, dysphagia, executive functions, and memory) have been added to the NIHSS. Moreover, two of the 15 original items (limb ataxia, sensory) have been modified to take into account bilateral damage. PML Score ranges from 0 to 60 points. This score is not yet validated at present.
(DOC)

Table S2 **Review of previous studies that reported effects of combination antiretroviral therapy on HIV-related PML: characteristics of patients at PML diagnosis.** Results are expressed as median, (range) or [IQR]. EDSS, Expanded Disability Status Score; KPS, Karnofsky Performance Score; NA, not available; ud, undetectable.
(DOC)

Checklist S1 Consort Statement 2001 Checklist for the paper entitled “Improved Survival of HIV-1-Infected Patients with Progressive Multifocal Leukoencephalopathy Receiving Early 5-Drug Combination Antiretroviral Therapy”.
(DOC)

Protocol S1 Short version of the protocol of the ANRS 125 Trial “Early Intensification of Antiretroviral Therapy Including Enfuvirtide in HIV-1-Related Progressive Multifocal Leucoencephalopathy”.
(DOC)
