# Supplementary material for: Improved Survival of HIV-1-Infected Patients with Progressive Multifocal Leukoencephalopathy Receiving Early 5-Drug Combination Antiretroviral Therapy
Source: PLoS One. 2011 Jun 30;6(6):e20967. doi: 10.1371/journal.pone.0020967 (PMC3127950; doi:10.1371/journal.pone.0020967)
Supplement: Table S1 — The PML Score is a clinical assessment tool adapted from the NIH Stroke Scale (NIHSS, Brott et al. Stroke 1989). To evaluate and document neurological status in PML patients, four items (vestibular function, dysphagia, executive functions, and memory) have been added to the NIHSS. Moreover, two of the 15 original items (limb ataxia, sensory) have been modified to take into account bilateral damage. PML Score ranges from 0 to 60 points. This score is not yet validated at present. (DOC) [file pone.0020967.s001.doc]

**Table S1 : PML Score**

| **Instructions** | **Scale Definition** | **Score** |
| --- | --- | --- |
| **1a. Level of Consciousness:**  The investigator must choose a response if a full evaluation is prevented by such obstacles as an endotracheal tube, language barrier, orotracheal trauma/bandages. A 3 is scored only if the patient makes no movement (other than reflexive posturing) in response to noxious stimulation. | 1. **Alert**; keenly responsive. 2. **Not alert**; but arousable by minor stimulation to obey, answer, or respond. 3. **Not alert**; requires repeated stimulation to attend, or is obtunded and requires strong or painful stimulation to make movements (not stereotyped). 4. Responds only with reflex motor or autonomic effects or totally unresponsive, flaccid, and areflexic. |  |
| **1b. LOC Questions:**  The patient is asked the month and his/her age. The answer must be correct - there is no partial credit for being close. Aphasic and stuporous patients who do not comprehend the questions will score 2. Patients unable to speak because of endotracheal intubation, orotracheal trauma, severe dysarthria from any cause, language barrier, or any other problem not secondary to aphasia are given a 1. It is important that only the initial answer be graded and that the examiner not "help" the patient with verbal or non-verbal cues. | 1. **Answers** both questions correctly. 2. **Answers** one question correctly. 3. **Answers** neither question correctly. |  |
| **1c. LOC Commands:**  The patient is asked to open and close the eyes and then to grip and release the non-paretic hand. Substitute another one step command if the hands cannot be used. Credit is given if an unequivocal attempt is made but not completed due to weakness. If the patient does not respond to command, the task should be demonstrated to him or her (pantomime), and the result scored (i.e., follows none, one or two commands). Patients with trauma, amputation, or other physical impediments should be given suitable one-step commands. Only the first attempt is scored. | 1. **Performs** both tasks correctly. 2. **Performs** one task correctly. 3. **Performs** neither task correctly**.** |  |
| **2. Best Gaze:**  Only horizontal eye movements will be tested. Voluntary or reflexive (oculocephalic) eye movements will be scored, but caloric testing is not done. If the patient has a conjugate deviation of the eyes that can be overcome by voluntary or reflexive activity, the score will be 1. If a patient has an isolated peripheral nerve paresis (CN III, IV or VI), score a 1. Gaze is testable in all aphasic patients. Patients with ocular trauma, bandages, pre-existing blindness, or other disorder of visual acuity or fields should be tested with reflexive movements, and a choice made by the investigator. Establishing eye contact and then moving about the patient from side to side will occasionally clarify the presence of partial gaze palsy. | 1. **Normal** 2. **Partial gaze palsy:** gaze is abnormal in one or either eyes, but forced deviation or total gaze paresis is not present. 3. **Forced deviation,** or total gaze paresis not overcome by the oculocephalic maneuver. |  |
| **3. Visual:**  Visual fields (upper and lower quadrants) are tested by confrontation, using finger counting or visual threat, as appropriate. Patients may be encouraged, but if they look at the side of the moving fingers appropriately, this can be scored as normal. If there is unilateral blindness or enucleation, visual fields in the remaining eye are scored. Score 1 only if a clear-cut asymmetry, including quadrantanopia, is found. If patient is blind from any cause, score 3. Double simultaneous stimulation is performed at this point. If there is extinction, patient receives a 1, and the results are used to respond to item “extinction and inattention”. | 1. **No visual loss.** 2. **Partial hemianopia.** 3. **Complete hemianopia.** 4. **Bilateral hemianopia** (blind including cortical blindness) |  |
| **4. Facial Palsy:**  Ask – or use pantomime to encourage – the patient to show teeth or raise eyebrows and close eyes. Score symmetry of grimace in response to noxious stimuli in the poorly responsive or non-comprehending patient. If facial trauma/bandages, orotracheal tube, tape or other physical barriers obscure the face, these should be removed to the extent possible. | 1. **Normal** symmetrical movements. 2. **Minor paralysis (**flattened nasolabial fold, asymmetry on smiling). 3. **Partial paralysis** (total or near-total paralysis of lower face). 4. **Complete paralysis of one or both sides** (absence of facial movement in the upper and lower face) |  |
| **5. Motor Arm:**  The limb is placed in the appropriate position: extend the arms (palms down) 90 degrees (if sitting) or 45 degrees (if supine). Drift is scored if the arm falls before 10 seconds. The aphasic patient is encouraged using urgency in the voice and pantomime, but not noxious stimulation. Each limb is tested in turn, beginning with the non-paretic arm. Only in the case of amputation or joint fusion at the shoulder, the examiner should record the score as untestable (UN), and clearly write the explanation for this choice.  **5a. Left Arm**  **5b. Right Arm** | 1. **No drift:** limb holds 90 (or 45) degrees for full 10 seconds. 2. **Drift:** limb holds 90 (or 45) degrees, but drifts down before full 10 seconds; does not hit bed or other support. 3. **Some effort against gravity:** limb cannot get to or maintain (if cued) 90 (or 45) degrees, drifts down to bed, but has some effort against gravity. 4. **No effort against gravity**: limb falls. 5. **No movement.**   **UN = Amputation** or joint fusion |  |
| **6. Motor Leg:**  The limb is placed in the appropriate position: hold the leg at 30 degrees (always tested supine). Drift is scored if the leg falls before 5 seconds. The aphasic patient is encouraged using urgency in the voice and pantomime, but not noxious stimulation. Each limb is tested in turn, beginning with the non-paretic leg. Only in the case of amputation or joint fusion at the hip, the examiner should record the score as untestable (UN), and clearly write the explanation for this choice.  **6a. Left Leg.**  **6b. Right Leg.** | 1. **No drift**: leg holds 30-degree position for full 5 seconds. 2. **Drift:** leg falls by the end of the 5-second period but does not hit bed. 3. **Some effort against gravity**: leg falls to bed by 5 seconds, but has some effort against gravity. 4. **No effort against gravity:** leg falls to bed immediately. 5. **No movement.**   **UN = Amputation** or joint fusion, |  |
| **7. Limb Ataxia:**  This item is aimed at finding evidence of a unilateral (or bilateral) cerebellar lesion. Test with eyes open. In case of visual defect, ensure testing is done in intact visual field. The finger-nose-finger and heel-shin tests are performed on both sides, and ataxia is scored only if present out of proportion to weakness. Ataxia is absent in the patient who cannot understand or is paralyzed. Only in the case of amputation or joint fusion, the examiner should record the score as untestable (UN), and clearly write the explanation for this choice. In case of blindness, test by having the patient touch nose from extended arm position.  **7a. Left Side.**  **7b. Right Side.** | 1. **Absent.** 2. **Present in one limb.** 3. **Present in two limbs.**   **UN = Amputation** or joint fusion. |  |
| **8. Sensory:**  Sensation or grimace to pinprick when tested, or withdrawal from noxious stimulus in the obtunded or aphasic patient. Only sensory loss attributed to PML is scored as abnormal and the examiner should test as many body areas (arms [not hands], legs, trunk, face) as needed to accurately check for hemisensory loss. A score of 2, “severe or total sensory loss,” should only be given when a severe or total loss of sensation can be clearly demonstrated. Stuporous and aphasic patients will, therefore, probably score 1 or 0. The patient with brainstem PML who has bilateral loss of sensation is scored 4. If the patient does not respond and is quadriplegic, score 4. Patients in a coma (item 1a=3) are automatically given a 4 on this item.  **8a. Left Side.**  **8b. Right Side** | 1. **Normal:** no sensory loss. 2. **Mild-to-moderate sensory loss:** patient feels pinprick is less sharp or is dull on the affected side; or there is a loss of superficial pain with pinprick, but patient is aware of being touched. 3. **Severe to total sensory loss**: patient is not being touched in the face, arm, and leg. |  |
| **9. Best Language**:  A great deal of information about comprehension will be obtained during the preceding sections of the examination. For this scale item, the patient is asked to describe what is happening in the attached picture, to name the items on the attached naming sheet and to read from the attached list of sentences. Comprehension is judged from responses here, as well as to all of the commands in the preceding general neurological exam. If visual loss interferes with the tests, ask the patient to identify objects placed in the hand, repeat, and produce speech. The intubated patient should be asked to write. The patient in a coma (item 1a=3) will automatically score 3 on this item. The examiner must choose a score for the patient with stupor or limited cooperation, but a score of 3 should be used only if the patient is mute and follows no one-step commands. | 1. **No aphasia:** normal. 2. **Mild-to-moderate aphasia:** some obvious loss of fluency or facility of comprehension, without significant limitation on ideas expressed or form of expression. Reduction of speech and/or comprehension, however, makes conversation about provided materials difficult or impossible. For example, in conversation about provided materials, examiner can identify picture or naming card content from patient’s response. 3. **Severe aphasia:** all communication is through fragmentary expression; great need for inference, questioning, and guessing by the listener. Range of information that can be exchanged is limited; listener carries burden of communication. Examiner cannot identify materials provided from patient response. 4. **Mute, global aphasia:** no usable speech or auditory comprehension |  |
| **10. Dysarthria:**  If patient is thought to be normal, an adequate sample of speech must be obtained by asking patient to read or repeat words from the attached list. If the patient has severe aphasia, the clarity of articulation of spontaneous speech can be rated. Only if the patient is intubated or has other physical barriers to producing speech, the examiner should record the score as untestable (UN), and clearly write an explanation for this choice. Do not tell the patient why he or she is being tested. | 1. **Normal.** 2. **Mild-to-moderate dysarthria:** patient slurs at least some words and, at worst, can be understood with some difficulty. 3. **Severe dysarthria:** patient's speech is so slurred as to be unintelligible in the absence of or out of proportion to any dysphasia, or is mute/anarthric.   **UN = Intubated** or other physical barrier |  |
| **11. Extinction and inattention** (formerly Neglect):  Sufficient information to identify neglect may be obtained during the prior testing. If the patient has a severe visual loss preventing visual double simultaneous stimulation, and the cutaneous stimuli are normal, the score is normal. If the patient has aphasia but does appear to attend to both sides, the score is normal. The presence of visual spatial neglect or anosagnosia may also be taken as evidence of abnormality. Since the abnormality is scored only if present, the item is never untestable. | 1. **No abnormality.** 2. **Visual, tactile, auditory, spatial, or personal inattention** or extinction to bilateral simultaneous stimulation in one of the sensory modalities. 3. **Profound hemi-inattention or extinction to more than one modality:** does not recognize own hand or orients to only one side of space. |  |
| **Additional Items** | |  |
| **12. Vestibular Function**:  This item is aimed at finding evidence of a central lesion of vestibular system. Patient will be asked about the presence of dizziness and oassociated symptoms. Bedside testing of vestibular function includes Romberg test, evaluation of gait (especially eyes closed) and observation for nystagmus (spontaneous, positional or after head shaking) | 1. **No abnormality.** 2. **Mild-to-moderate dizziness and nausea,** increasing with position changes. Minor nystagmus. 3. **Constant dizziness and vomiting.** Standing impossible.Severe nystagmus. |  |
| **13. Dysphagia:**  Swallowing liquids, foods, and saliva is tested with special attention to the occurrence of coughing or throat clearing during ingestion. | 1. **Normal swallowing.** 2. **Minimal to moderate dysphagia:** Difficulty to swallow liquids requiring precautions when feeding. 3. **Severe dysphagia:** serious troubles to swallow safely or completely unable to swallow requiring the cessation of all oral intake |  |
| **14. Executive Functions**:  Identification of executive dysfunction may be obtained during the preceding sections of the examination. For this scale item, the patient is asked to perform some tests as the Luria hand sequence and the clock drawing test. | 1. **No abnormality.** 2. **Mild disturbance,** with no interference with every day functioning. 3. **Moderate disturbance:** interference in daily-living activities and/or social functioning. 4. **Severe dysexecutive syndrome,** with marked interference in daily-living activities; at most, total dependence. |  |
| **15. Memory**:  Identification of memory dysfunction may be obtained during the preceding sections of the examination. For this scale item, the patient is asked to perform the five-word test. | 1. **No abnormality.** 2. **Mild disturbance,** with no interference with every day functioning. 3. **Moderate disturbance:** Swallowing very difficult requiring the cessation of all oral intake 4. **Severe amnesia,** with marked interference in daily-living activities; at most total dependence**.** |  |
