# Supplementary material for: Improved Survival of HIV-1-Infected Patients with Progressive Multifocal Leukoencephalopathy Receiving Early 5-Drug Combination Antiretroviral Therapy
Source: PLoS One. 2011 Jun 30;6(6):e20967. doi: 10.1371/journal.pone.0020967 (PMC3127950; doi:10.1371/journal.pone.0020967)
Supplement: Table S2 — Review of previous studies that reported effects of combination antiretroviral therapy on HIV-related PML: characteristics of patients at PML diagnosis. Results are expressed as median, (range) or [IQR]. EDSS, Expanded Disability Status Score; KPS, Karnofsky Performance Score; NA, not available; ud, undetectable. (DOC) [file pone.0020967.s002.doc]

**Table S2. Review of previous studies that reported effects of combination antiretroviral therapy on HIV-related PML: characteristics of patients at PML diagnosis.** Results are expressed as median, (range) or [IQR]. EDSS, Expanded Disability Status Score; KPS, Karnofsky Performance Score; NA, not available; ud, undetectable.

| **References** | **Study period** | **Samples** | **ART naive (%)** | **CD4+ T cells** (per µL) | **Plasma HIV RNA**  *(log_10_ copies/mL)* | **CSF JCV DNA**  *(log_10_ copies/mL*) | **Pre-treatment disability** |
| --- | --- | --- | --- | --- | --- | --- | --- |
| Clifford et al. (11) | Before 1998 | 25 | 40% | 104 (4-1030)] | 3.5 (ud-6.2) | NA | NA |
| Gasnault et al. (12) | 1995-1998 | 23 | 48% | 84 [24-134] | 4.8 [4.0-5.5] | 4.5 [3.9-5.2] | EDSS  4.5 [4.0-6.0] |
| De Luca et al. (13) | 1993-1999 | 32 | NA | 38 [15-77] | 4.8 [3.5-5.5] | 3.3 [3.2-4.3] | KPS  50 (20-80) |
| Cinque et al. (19) | 1996-2000 | 27 | 37% | R : 45 (8-397)  NR : 60 (6-418) | R : 4.9 (3.3-6.0)  NR : 4.8 (ud-6.0) | NA | NA |
| Antinori et al. (14) | 2000-2002 | 101 | 17% | 88 [27-176] | 4.8 [3.7-5.3] | NA | NA |
| De Luca et al. (18) | 1996-2004 | 370 | 33% | 73 [29-157] | 5.0 [4.0-5.4] | 3.7 [3.2-4.8] | KPS  50 [40-70] |
| Falco et al. (25) | 2002-2006 | 61 | 60% | 90 [27-177] | 5.0 [3.9-5.5] | NA | NA |
| Engsig et al. (3) | 1995-2006 | 47 | 75% | 50 [27-160] | 4.9 [3.7-5.6] | NA | NA |
