# Supplementary material for: Improved Survival of HIV-1-Infected Patients with Progressive Multifocal Leukoencephalopathy Receiving Early 5-Drug Combination Antiretroviral Therapy
Source: PLoS One. 2011 Jun 30;6(6):e20967. doi: 10.1371/journal.pone.0020967 (PMC3127950; doi:10.1371/journal.pone.0020967)
Supplement: Protocol S1 — Short version of the protocol of the ANRS 125 Trial “Early Intensification of Antiretroviral Therapy Including Enfuvirtide in HIV-1-Related Progressive Multifocal Leucoencephalopathy”. (DOC) [file pone.0020967.s004.doc]

**Early intensification of antiretroviral therapy including enfuvirtide in HIV-1-related progressive multifocal leucoencephalopathy (ANRS 125)**

**Sponsored by:** French National Agency for Research on AIDS and Viral Hepatitis

**Collaborators:** Hoffmann-La Roche, Gilead Sciences

**Information provided by:** French National Agency for Research on AIDS and Viral **Hepatitis**

**ClinicalTrials.gov Identifier:** NCT00120367

**Purpose**

Progressive multifocal leucoencephalopathy (PML) is a rare infectious disease of the brain, provoked by the JC virus. It usually occurs in subjects with impaired immune system as during HIV infection. To date, there is no specific antiviral treatment susceptible to cure PML. But in the setting of HIV-related PML, it was shown that combination antiretroviral therapy allows a restoration of the immune system and then might stop the progression of PML.

The objective of this study is to appreciate the supplementary efficiency brought by an association of more powerful antiretroviral molecules including enfuvirtide (FUZEON®) on the evolution of PML in HIV-1-infected patients. This research program will involve 30 patients in several centres in France. All the patients who will participate will receive enfuvirtide during 6 months in association with a combination of two or more potent antiretroviral drugs. The total duration of follow-up for a patient will be of 1 year.

Another aim of this study is to assess on treatment the evolution of immunological and virological factors related to JCvirus the causative agent of PML.

*Study Type*: Interventional

*Study Phase:* Phase II

*Study Design:* Treatment, Non-Randomized, Single Group Assignment, Open Label, Multicentre, Efficacy Study.

*Official Title:* Early intensification of combination antiretroviral therapy including enfuvirtide in the treatment of progressive multifocal leucoencephalopathy during HIV-1 infection. The ANRS 125 trial.

**Further Study Details:**

*Primary Outcome Measure:*

- Estimation by the method of Kaplan-Meier of the rate of survival at M12

*Secondary Outcome Measures:*

- Rate of survival and functional score (Modified Rankin Outcome Scale) at M12
- Evolution of the JC viral load in the CSF and percentage of patients with JC virus clearance of the CSF at M3 and M6
- Evolution of the CD4 and CD8 T cells sub- populations and of the antivirus JC specific T cell responses

*Expected Total Enrollment:* 30

*Study Start Date:* April 2005

*Study Completion Date:* December 2007

**Detailed Description:**

The aim of this open-label multicentre study is to estimate the effect of an early therapy intensification based on potent antiretroviral combination including enfuvirtide on survival in patients with HIV-1-related PML.

To demonstrate an expected one-year survival rate of 70%, significantly higher than 45% (the average of the one-year survival rate observed in previous reports), the inclusion of 24 patients is necessary. At last, 30 patients will be recruited towards the risk estimated at 25% of invalid inclusion.

Patients will be included on the following inclusion criteria : HIV-1 documented by Western Blot, clinical and radiological (MRI) evidence of active PML with clinical evolution (or deterioration) for less than 90 days, documentation of PML diagnosis for less than 30 days at the inclusion, informed consent (patient or confidence surrogate if decision making incapacity). Exclusion criteria will be the following: age less than 18-year-old; concomitant opportunistic infection of the central nervous system; pregnancy - feeding; co-infection by the HIV-2; history of immunotherapy (interleukin 2, alpha-interferon) or of treatment by enfuvirtide; history of treatment by cidofovir; contra-indication to receive enfuvirtide.

An independent committee will meet regularly to estimate the validity of PML diagnosis in included patients.

All the patients receive enfuvirtide during the first 6 months in association with a combination of two or more antiretroviral drugs which will be pursued during the next 6 months till the end of the study (M12). Antiretroviral-naïve patients will receive lopinavir/ritonavir, efavirenz, and tenofovir/emtricitabine (under the shape of TRUVADA®). For pretreated patients, antiretroviral combination will be chosen in every case on the basis of the therapeutic history and of the viral genotypes of resistance. Such association will contain at least two antiretroviral molecules, issued from two different families among the three following ones (nucleoside inhibitors of the reverse transcriptase, non-nucleoside inhibitors of the reverse transcriptase, protease inhibitors).

**Eligibility**

*Ages Eligible for Study:* 18 Years and older

*Genders Eligible for Study:* Both

*Accepts Healthy Volunteers:* No

***Criteria***

*Inclusion Criteria:*

- Men and non pregnant women
- 18 years of age and older
- Have confirmed laboratory diagnosis of HIV infection
- Presenting with a clinical history of active PML evolving (or continuing to deteriorate) for less than 90 days
- Diagnosis of PML documented for less than 30 days at the inclusion by cerebral imaging (MRI) AND the absence of another demonstrated etiology AND the detection of JCV DNA in the CSF by qualitative PCR
- Signed written inform consent

*Exclusion Criteria:*

- Concomitant opportunistic infection of the central nervous system
- Pregnancy – feeding
- Co-infection with the HIV-2
- History of immunotherapy including interleukine 2 or alpha-interferon
- History of treatment including enfuvirtide or cidofovir
- Contra-indication to receive enfuvirtide.

**Location and Contact Informations**

Please refer to this study by its ClinicalTrials.gov identifier: NCT00120367

***France***

Jacques Gasnault, MD, 00 331 45 21 63 54, jacques.gasnault@bct.aphp.fr

Service de Médecine Interne et Maladies Infectieuses, Hôpital Bicêtre, Le Kremlin Bicêtre, 94270, France

***Sponsors and Collaborators***

French National Agency for Research on AIDS and Viral Hepatitis

Hoffmann-La Roche

Gilead Sciences

***Investigators***

*Principal Investigator:* Jacques Gasnault, MD, Hôpital Bicêtre, Le Kremlin Bicêtre, 94270, France

*Study Chair:* Dominique Costagliola, Inserm U943, Paris 75625 Cedex 13, France

**Abstract for patients**

Progressive multifocal leucoencephalopathy (PML) is a rare infectious disease of the brain, provoked by the JC virus. It usually occurs in subjects with impaired immune system as during HIV infection. To date, there is no specific antiviral treatment susceptible to cure PML. But it was shown in the setting of HIV-related PML, that combination antiretroviral therapy allows a restoration of the immune system and then might stop the progression of PML.

The objective of this study is to appreciate the supplementary efficiency brought by an association of more powerful antiretroviral molecules including FUZEON® on the evolution of PML. This research program will involve 30 patients in several centres in France. All the patients who will participate will receive FUZEON® during 6 months in association with a combination of two or more potent antiretroviral drugs. The total duration of follow-up for a patient will be of 1 year.
